# Climate, Weather, and Ecology in Evaluation of High Latitude Spring Wheat Breeding Sites and Germplasm

**DOI:** 10.3390/plants14213256

**Published:** 2025-10-24

**Authors:** Alexey Morgounov, Mikhail Divashuk, Anastasia Chernook, Daniil Ulyanov, Oleg Kuzmin, Ekaterina Shreyder, Nadya Bondarenko, Klavdiya Volokitina, Anastasia Kazak, Daniyar Tajibayev, Vladimir Shamanin

**Affiliations:** 1 All-Russia Research Institute of Agricultural Biotechnology, Moscow 127550, Russia; divashuk@gmail.com (M.D.); irbis-sibri@yandex.ru (A.C.); uldas1508@gmail.com (D.U.); 2 Agrotechnology Department, Omsk State Agrarian University Named After Stolypin, Omsk 644008, Russia; og.kuzmin@omgau.org (O.K.); vp.shamanin@omgau.org (V.S.); 3 Chelyabinsk Scientific Research Institute of Agriculture, Timiryazevo, Chelyabinsk 456404, Russia; shreyder11@mail.ru (E.S.); bondarenko_np61@mail.ru (N.B.); 4 Kurgan Seeds Research Center, Kurgan 640000, Russia; klava.volokitina@ya.ru; 5 Agricultural Institute, University of Tyumen, Tyumen 625003, Russia; kazaknastenka@rambler.ru; 6 Kazakh Research Institute of Agriculture and Plant Growing, Almaty 050010, Kazakhstan; daniyar.taj@gmail.com

**Keywords:** *Triticum aestivum* L, breeding, genotype—environment interaction, weather, grain yield

## Abstract

The Ural Mountains in the Western Siberia region cultivate over 3.5 M ha of short season spring wheat, with an average grain yield of 1.6–2.0 t/ha. The study focus was the analysis of climate change and weather effects on spring wheat yields from 2001 to 2024 and on genotype–environment interactions in the Kazakhstan–Siberia Spring Wheat Improvement network (KASIB) trials from 2019 to 2024. Climate change has the tendency to gradually reduce precipitation and increase air temperatures, which negatively affect spring wheat yields. Based on regional yield and weather, the region was divided into subregions: Tyumen in the North with a high yield; Chelyabinsk with lower precipitation and a lower grain yield; and Omsk and Kurgan were similar in most years. Environments at the four breeding programs (Chelyabinsk Agricultural Research Institute, Kurgan Seeds, and Omsk and Tyumen State Agrarian Universities) did not fully reflect the target production areas due to a very high yield gap and lack of association between the research and production yields. Genotype–environment interaction analysis showed that the Tyumen site had the highest yield and best discriminating ability, while Chelyabinsk best represented the whole target region. Most of the highest yielding material in KASIB trials originated from outside of the region. Spring wheat breeding programs in the region ought to improve to maintain a competitive edge.

## 1. Introduction

Wheat is an essential global crop commodity grown across continents, providing daily food to billions of people. According to the FAO database (https://www.fao.org/faostat (accessed on 9 September 2025)), the global area under wheat was 220 M ha, with a total production of 799 M tons and an average grain yield of 3.62 t/ha. The Russian Federation is one of the main wheat producers, accounting for 13.1% of the area (28.8 M ha) and 11.4% production (91.5 M t). A substantial amount of wheat is exported (31.6 M t in 2023), contributing to regional and global food security. The current situation and perspectives of agricultural production in Russia are presented in a book titled *Russia’s Role in the Contemporary International Agri-Food Trade System* [1].

The structure of wheat production in Russia depends on geography, environmental conditions and cropping system. The main production region is in the fertile South of European part of the country (Krasnodar, Rostov and Stavropol regions) with dominant winter wheat yielding above 5–6 t/ha [2]. Central part and Volga region cultivate both winter and spring wheat while moving to the East (Ural Mountains and Western Siberia) the share of spring wheat increases and almost entirely dominates production.

The spring wheat belt, which stretches from the Ural Mountains through Northern Kazakhstan and Western Siberia, collectively cultivates over 15 M ha of wheat. Spring wheat dominates the production in this belt, covering over 50–60% of arable land. It is cultivated in rotation with other cereals (barley), oil crops (sunflower, linseed, and rapeseed), and legumes (dry peas and lentil) [3]. The main limiting environmental factor in the region is the availability of moisture, with an annual precipitation of 300–450 mm. Extensive spring wheat production technology is practiced with limited, if any, fertilizers, and crop protection is focused on weeds and diseases. As a result, the yields are in a range of 1.3–1.8 t/ha, well below the potential [4].

Spring wheat cultivars represent an important technological component. The majority of the varieties grown in the region of Ural–North Kazakhstan–Siberia represent the tall, extensive type. New, shorter stature European cultivars are being successfully introduced and competing with local material [5]. Breeding programs in the region are united through the Kazakhstan–Siberia Network on Spring Wheat Improvement (KASIB), established in 2000 [6]. The network comprises over 12 spring wheat breeding programs from two countries and conducts biannual cooperative trials. Every two years, the cooperators submit 2–3 new varieties or breeding lines, which are tested in replicated field trials in all sites, with data combined, analyzed, and distributed within the network. The trial assists in the identification of superior germplasm through the analysis of multilocational performance. KASIB trials data accumulated over the years represent a valuable source for genotype x environment interaction analysis, genetic gains, and other studies [7,8,9].

Climate change associated with crop production challenges represents a worldwide concern. According to the annual climate report in Russia [10], warming has been observed throughout Russia in all seasons. The growth rate of the average annual temperature in Russia was +0.50 °C/10 years, while warming in spring was the highest (0.63 °C/10 years), especially in Western Siberia (+0.72 °C/10 years). The trend towards an increase in annual precipitation amounts to 1.8% per 10 years. The trend exceeds 5%/10 years in some regions of Siberia and the Far East. The period of active vegetation (T > 10 °C) of agricultural crops increased the duration of the growing season up to 4–5 days/10 years. There is a trend towards an increase in summer precipitation in Siberia and the Far East at a rate of ~5 and ~9 mm/10 years, respectively. Despite the positive trends of temperature and precipitation increases, there is also a high probability of droughts across all wheat production areas.

Several studies have been devoted to the effect of climate change on spring wheat in Russia. Goncharov et al. [11] analyzed the spring cereals yield tendency from 1990 to 2020 and concluded that an extended vegetation period driven by climate change will increase the yield by 10–12% in the short (2025–30) and medium (2045–50) term. The recent increase in grain production in Russia is not fully explained by the “weather-yield” crop models, which project only moderate yield increases in the twenty-first century [12]. There is evidence of a high contribution of agricultural reforms in yield improvements. Abys et al. [13] argue that Russia’s growth in wheat production is partly due to the replacement of spring by winter wheat due to the warming climate and partly due to production intensification. The yields of spring wheat in the main production belt of Ural–North Kazakhstan–Siberia remain considerably lower compared to similar cropping systems in North America [14]. There is lack of knowledge linking climate change with production yields and the wheat breeding framework.

The current study focused on four regions in the Ural Mountains and Western Siberia production zone (Chelyabinsk, Kurgan, Omsk, and Tyumen), with an average grain yield of 1.5–1.8 t/ha. They collectively cultivate 3.5 M ha of spring wheat, and each region houses spring wheat breeding programs. The study focus was the analysis of climate change effects on regional wheat yields from 2001 to 2024, the related analysis of spring wheat agronomic trait variations in KASIB trials from 2019 to 2024, and comprehensive genotype–environment interaction analysis to assist breeding programs in the development of perspective strategies. The study’s important outcome was the characterization of breeding sites and identification of superior germplasm for use in breeding and production.

## 2. Materials and Methods

### 2.1. Weather and Production Data Sources

The study utilized monthly weather data (precipitation and air temperature) from meteo stations situated in Chelyabinsk, Kurgan, Omsk, and Tyumen from 2001 to 2024. The data was accessible from the database of All-Russian Research Institute of Hydroclimatic Information [15] (http://meteo.ru/data/ (accessed on 20 August 2025)). Spring wheat annual area and grain yield data for the four study regions from 2001 to 2024 was obtained from consecutive Russian Statistical Agency electronic publications of “Russia agriculture” (https://rosstat.gov.ru/folder/210/document/13226 (accessed on 15 August 2025)). The weather and yield data was combined in an Excel file for analysis. Evaluation of climate change over 24 years of study was conducted using mean values for weather parameters for three 8-year periods: 2001–08; 2009–16; and 2017–24.

### 2.2. Experimental Sites

**Figure 1:**
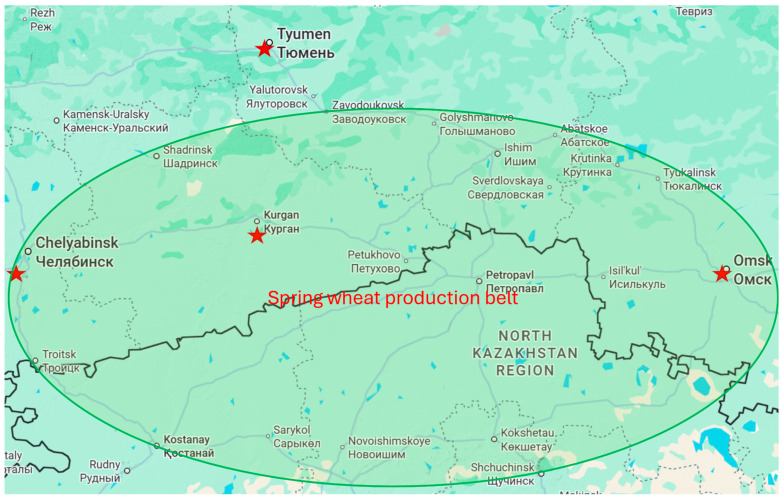
Map of Ural Mountains–North Kazakhstan–Western Siberia crop production zone, with locations of the four spring wheat breeding programs included in the study.

KASIB trials were conducted at four spring wheat breeding programs: Chelyabinsk Scientific Research Institute of Agriculture (Chelyabinsk ARI), private company Kurgan Seeds Research Center, Omsk State Agrarian University, named after Stolypin (Omsk SAU), and Northern Trans-Ural State Agrarian University in Tyumen (Tyumen SAU). The breeding program sites are situated above 55 °N and east of 60 °N, which separates Europe from Asia (Table 1, Figure 1). The region’s ecology is steppe in the south, transforming to forest–steppe and forest zone with the move to the north. The distance from Chelyabinsk to Kurgan is 270 km, and from Kurgan to Omsk it is 550 km. Tyumen is 200 km north from Kurgan. The soil of experimental sites represented by different types of chernozem: leached in Omsk and Chelyabinsk, regular in Kurgan, and meadow chernozem in Tyumen. The organic matter content in the soils varied withing 3.5–5.0%. The sites represent high-latitude wheat production environments similar to Canada [16].

Annual precipitation and air temperature for experimental sites is presented in Table 1. Average annual precipitation for 24 years was 464 mm in Tyumen, 20% higher compared to Chelyabinsk and Kurgan and 11% higher than in Omsk. The difference in annual air temperature between contrasting sites (Chelyabinsk–Omsk) was 1.2 °C. The differences in weather parameters during the spring wheat growing season (May–August) were even higher: 28.7% for precipitation (Chelyabinsk–Tyumen) and 11.1% for air temperature. Overall, the four regions are different and also representative of this spring wheat production zone.

### 2.3. KASIB Spring Wheat Germplasm, Field Trials Methodology, and Statistics

Three KASIB trials were included in the study: 2019–20 KASIB comprised 52 entries, 2021–22—42 entries, and 2023–24—45 entries (Table S1). The germplasm was represented by the recent cultivars and breeding lines from KASIB cooperators in Kazakhstan (6–7 breeding programs, 40–45% of germplasm) and Russia (9–10 breeding programs, 55–60% of germplasm). The trials included five checks: Pamyaty Aziyeva (early maturing check), Tertsiya (leaf rust resistance check), Astana 2 (intermedium maturity check), Omskaya 35 (late maturity check), and Saratovskaya 29 (long-term historical check).

The trials’ agronomy followed established local practices: black fallow as preceding crop, planting in mid-May and harvesting in late August or September, and weed control with herbicides; no fertilizers or disease protection was used. Trials were planted using RCBD design with 3–5 m^2^ plots and 2–3 replications depending on the site. Four traits were recorded at all trials: number of days from germination to heading (DH), plant height (PH), 1000 kernel weight (TKW), and grain yield. Methodology described in CIMMYT Phenotyping Manual [17] was used for traits evaluation.

Statistical analysis included calculations of averages and standard errors and coefficients of correlation in MS Excel. Biplot analysis was employed to investigate the relationships between breeding sites, weather conditions, adaptation, and yield traits. ANOVA was conducted for adaptation and yield traits, and LSD calculations were used to evaluate the significance of differences between genotypes. Complementing the ANOVA/mixed effects workflow, we executed an iterative GGE/stability analysis program: data ingestion and cleaning, GGE modeling by KASIB groups, variance decomposition and ANOVA, computation of stability metrics with genotype ranking, and correlation and clustering of environments. R Studio version 3.4 [18] 2024.12.1 was utilized for these analyses.

## 3. Results

### 3.1. Climate Change, Weather, and Spring Wheat Grain Yield Variation in the Target Regions

#### 3.1.1. Wheat Production, Climate Change, and Weather Variation at Four Study Regions in 2001–24

**Figure 2:**
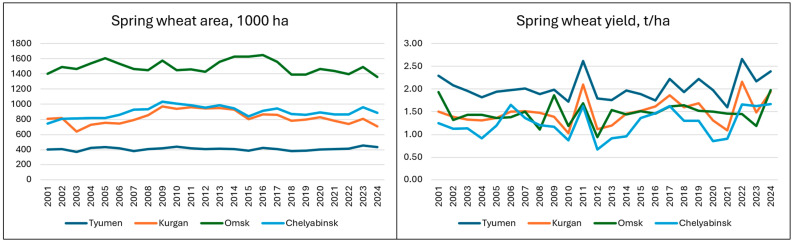
Dynamic of spring wheat area and grain yield in four regions in 2001–2024.

The total arable land in the four study regions was relatively stable from 2001 to 2024 and accounted for 7.2–7.4 M ha. The spring wheat area was also stable, varying from 3.5 to 3.8 M ha (Figure 2). The Omsk region dominated spring wheat production with a 2021–24 average area of 1.43 M ha, followed by Chelyabinsk (0.90 M ha), Kurgan (0.77 M ha), and Tyumen (0.42 M ha). The yearly variation in wheat area was 4.8% for Tyumen and 10.6% for Kurgan. The share of spring wheat in arable land demonstrated a slight declining tendency, being 51.8% in 2001–05 and 48.5% in 2021–24 across four regions. Overall, spring wheat represents a major established crop in this agroecological region.

Grain yield demonstrated a much higher yearly variation (Figure 2). The highest average spring wheat yield for 2001–24 was in Tyumen—2.03 t/ha (CV = 13.2%), followed by Kurgan—1.50 t/ha (CV = 19.4%), Omsk—1.48 t/ha (CV = 16.4%), and Chelyabinsk—1.28 t/ha (CV = 21.2%). The lowest grain yield in the Chelyabinsk region was associated with the highest variation. Yearly yield variation in the four regions follows a similar pattern with the highest and lowest yields in the same years.

**Figure 3:**
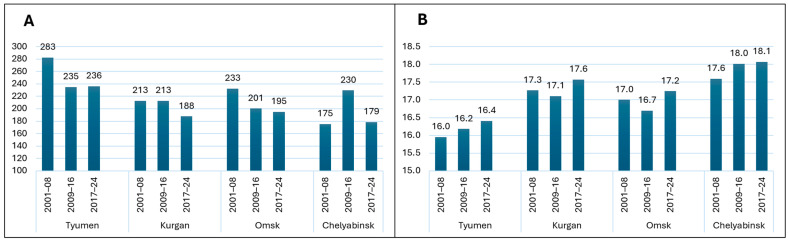
Changes in seasonal (May–August) precipitation (**A**) and air temperature (**B**) for three 8-year periods from 2001 to 2024 in four study regions.

Evaluation of climate change showed that almost all regions, with the exception of Chelyabinsk, experienced a seasonal (May–August) rainfall reduction in 2017–24 compared to 2001–08. It varied from 25 mm (11.3%) in Kurgan to 47 mm (16.6%) in Tyumen (Figure 3, Tables S2 and S3). The yearly figures also showed a precipitation reduction in Tyumen, Kurgan, and Omsk in 2017–24 compared to 2001–08 (Table S2). Interestingly, the period from 2009 to 2016 witnessed a slight increase in yearly precipitation in Chelyabinsk, indicating the cyclical nature of climate change. Air temperature changes were opposite to precipitation and increased slightly in 2017–24 compared to 2001–08. The increase was in the range of 0.2–0.5 °C, which may be substantial for wheat.

Coefficients of correlation between weather parameters and years were calculated for yearly, seasonal, and monthly precipitation and air temperature values from 2001 to 2024. For precipitation, only one significant value was identified: the correlation between rainfall in July in Tyumen and years was −0.41, significant at *p* over 95%. For temperature, there was a significant positive correlation between the April temperature and years for all four regions. The correlation values were in the range 0.43–0.52. For the Omsk region, significant correlation was also found between the July temperature and years.

Climate change during the period of 2001–24 was expressed in the reduction in annual and seasonal precipitation by 11–17%. Precipitation reduction was specifically proven for July in Tyumen through the significant negative correlation with years. The air temperature had been gradually increasing, with a significant rise in April across all four regions.

#### 3.1.2. Variation in Spring Wheat Grain Yield and Its Relationship to Weather

**Figure 4:**
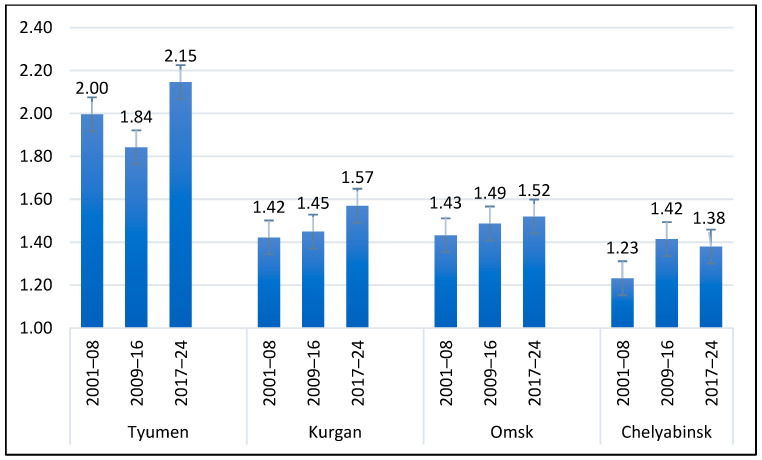
Changes in grain yield for three 8-year periods from 2001 to 2024 in four study regions.

Despite the negative tendencies of climate change in the study region, spring wheat grain yield had an increasing tendency during the period of 2001–2024 (Figure 4). The contribution of time periods and sites to variation was highly significant while their interaction was not. The yield gain of the 2017–24 period over 2001–08 was 12.2% for Chelyabinsk, 10.6% for Kurgan, 7.5% for Tyumen, and 6.3% for Omsk. The yield gain was not linear in Tyumen, where the intermediate period (2009–16) was lower, and in Chelyabinsk, where it was higher.

A significant positive correlation was recorded between the yield and rainfall in June (Tyumen, Kurgan, and Chelyabinsk), July (Omsk), and May–August (Kurgan, Omsk, and Chelyabinsk) (Table S4). Yield had significant negative correlation with the air temperature in May (Kurgan, Chelyabinsk), July (Omsk), and May–August (Tyumen, Kurgan, and Chelyabinsk). The changes in weather parameters in 2001–2024 in the study regions were supposed to have a negative effect on the yield. The increase in temperature and decrease in precipitation reduced the grain yield. However, the yield demonstrated some gains varying from 6 to 12% over 24 years. Technologies, cultivars, and policies contributed to yield gain in the region.

#### 3.1.3. Grouping of Regional Environments Based on Weather Parameters and Grain Yield

**Figure 5:**
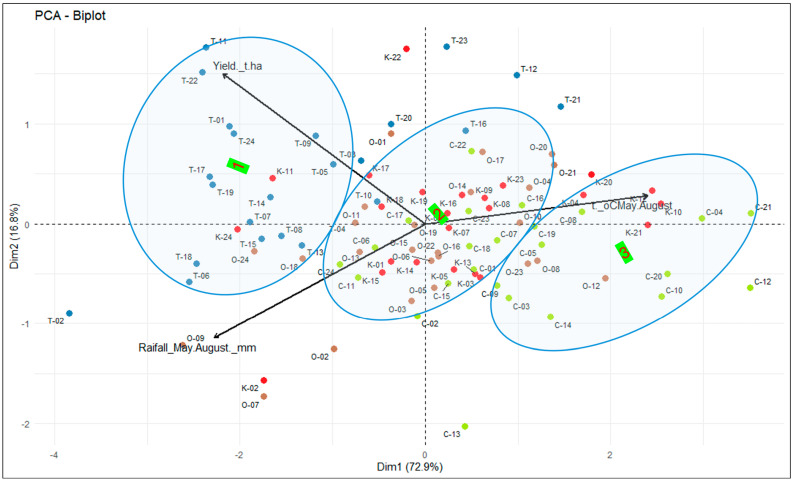
Biplot of four regions’ grain yields, seasonal precipitation, and air temperatures in 2001–24 (each region is designated by color and letter: C—Chelyabinsk, green; K—Kurgan, red; O—Omsk, brown; and T—Tyumen, blue, followed by a year).

The combination of the four regions’ yields and seasonal weather data for 24 years (96 data points total) was subjected to biplot analysis to delineate potential agroecological diversity and subregions within the whole region (Figure 5). The first principal component explains 72.9% of variation and the second explains 16.8%. The vectors of grain yield and seasonal rainfall are associated, while the air temperature vector has the opposite direction, indicating a negative relationship with these two traits. The majority of Tyumen environments–years were in cluster 1 along the yield vector, with two Kurgan and two Omsk environments. The largest cluster, cluster 2, primarily comprised Omsk and Kurgan environments with several Chelyabinsk years and only two Tyumen years. Cluster 3, along the temperature vector, was dominated by Chelyabinsk environments, with a few years from Omsk and Kurgan.

Overall, Tyumen represents quite the distinct subregion with higher spring wheat yields, higher seasonal rainfall, and lower air temperatures. The Chelyabinsk region is an opposite environment, with lower precipitation, higher temperatures, and a lower grain yield. In some years, it overlaps with Kurgan and Omsk but not Tyumen. Omsk and Kurgan environments are similar in most years and are sometimes also similar to Tyumen and Chelyabinsk. From a spring wheat breeding target viewpoint, Tyumen can be served by a breeding program targeting specific adaptation to this region. Omsk and Kurgan regions can be addressed by breeding programs developing germplasm with a broader adaptation to these two regions. The resulting cultivars may also be adapted to Chelyabinsk conditions which, in turn, require more specific adaptation to drier and hotter conditions.

### 3.2. Genotype–Environment Interaction in KASIB Spring Wheat Trials

#### 3.2.1. Variation in Agronomic and Adaptation Traits in KASIB Trials

The next step of the study was to analyze three biannual KASIB trials in four regions to evaluate the diversity and similarities of the breeding programs. The grain yield at the breeding institutions was substantially higher compared to the regional yields by 78–136% (Omsk–Chelyabinsk) (Table 2). The highest average yield for 2019–2024 was in Tyumen—4.14 t/ha, followed by Chelyabinsk (3.15 t/ha), Kurgan Seeds (2.92 t/ha), and Omsk (2.72 t/ha). The KASIB trial average grain yield variation at each breeding program was very high (CV = 31–52%), normally twice as high compared to production yields. Large differences between on-station and on-farm yields indicate a substantial yield gap and potential for productivity improvement. There is also misrepresentation of the target spring wheat area in the fields used for breeding purposes.

**Figure 6:**
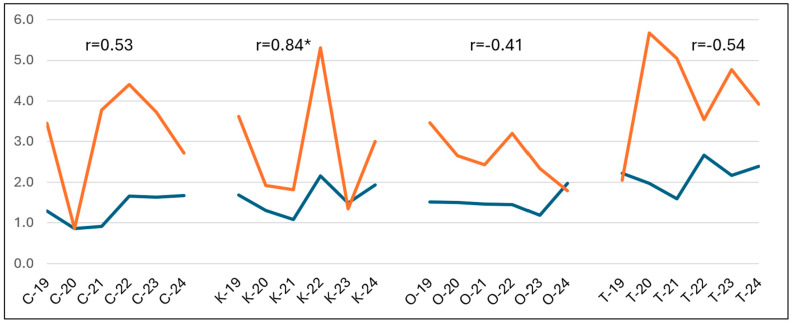
Variation in production (blue) and KASIB trials (orange) spring wheat yields across four regions (C—Chelyabinsk, K—Kurgan, O—Omsk, and T—Tyumen) in 2019–24.

The relationship between grain yield at KASIB sites and in the respective production regions in 2019–24, along with correlations between the two, is presented in Figure 6. This variation was positive, high, and significant in Kurgan (r = 0.84 *), insignificantly positive with average strength in Chelyabinsk (r = 0.53), and negative at two other sites. In Omsk and Tyumen, higher or lower production yields were not reflected at the research stations. In contrast, the yield variations were in opposite directions. The gap between the on-station and on-farm yields was minimal during the low-yielding years. Overall, this is another indication of misrepresentation of the target breeding environments by the respective breeding programs.

The rate of crop development and plant height are important traits defining adaptation to production environments. The difference between the sites for the duration period from germination to heading was 4.8 days and for height it was 10.4 cm (Table 2). Spring wheat in Kurgan was the shortest (69.3 cm), with the longest period of germination–heading (44.9 days). Tyumen wheat was faster developing and tallest among all sites. An important yield component, the 1000 kernel weight was highest in Omsk (39.2 g) and lowest in Kurgan (33.1 g).

The effect of variations in two adaptation traits on grain yield is an important source of genotype–environment interaction. Genotypic correlations were calculated for each KASIB trial at each site between DH, PH, TKW, and the grain yield (Table S6). The number of days to heading showed a significant positive correlation with the grain yield in Chelyabinsk (2021), Kurgan (2022), and Tyumen (2020). Later maturing genotypes generally tend to outperform the earlier maturing ones, especially under favorable conditions. These significant correlations were recorded in years with a high yield. Plant height had a significant positive correlation with yield in Chelyabinsk (2020, 2021), Kurgan (2020), and Tyumen (2019, 2020). TKW significantly contributed to the yield in Chelyabinsk (2021, 2024), Kurgan (2022), and Tyumen (2024). Obviously, all four breeding sites have a specific relationship between the adaptation traits and grain yield, resulting in genotype–environment interactions.

Wheat diseases and leaf rust specifically affected the spring wheat yield in the study region. Leaf rust was observed in Omsk in 2024 and in Chelyabinsk every year from 2021 to 2024, with susceptible entries reaching an 80–100% severity. Grain yield and leaf rust severity had a significant negative correlation in Chelyabinsk in 2022 (−0.59 ***), in 2024 (−0.48 ***), and in Omsk in 2024 (−0.39 **). Obviously, favorable years with higher rainfall caused leaf rust, which reduced the grain yield. The frequency of leaf rust-resistant germplasm varied from 20 to 35% depending on the trial.

#### 3.2.2. Genotype–Environment Interactions in KASIB Trial

ANOVA results for the grain yield are presented in Table S7. The most significant sources of variation in all three trials were sites and years, while the effect of genotypes was significant only in KASIB 2019–20. The effect of the genotype–environment interaction was non-significant. This is due to a relatively large number of genotypes with a similar grain yield and likely limited genetic diversity. However, from the breeding perspective, genotype–environment interactions can be estimated by the correlation between the same set of germplasm in different sites or at the same site in different years (Table 3). Interestingly, only one trial out of three had significant correlations in germplasm yield performance between years at the same breeding site. This means that the yield and ranking of genotypes at the same site in two subsequent years did not match in two KASIB trials out of three. The genotypes’ yield performances at Chelyabinsk ARI correlated well with all three sites’ performances: correlations of 0.49–0.73 with Kurgan, 0.46–0.66 with Omsk, and 0.54–0.60 with Tyumen. Kurgan–Tyumen and Omsk–Tyumen had the lowest correlations between respective germplasm yield performances.

This correlation analysis defined the presence of genotype–environment interactions between the years at the same site and between the sites within one trial. It appears that the germplasm performance at Chelyabinsk was relatively well related to the performance at all other sites, while Tyumen is less related to Kurgan and Omsk.

#### 3.2.3. Evaluation of the Breeding Sites for Representation and Discriminating Ability

**Figure 7:**
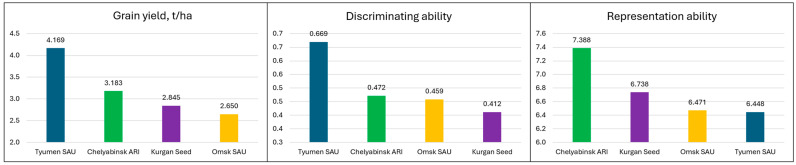
GGE parameters characterizing breeding sites.

GGE analysis results, including average values for grain yield and breeding, cites characteristics across all three KASIB trials, which are presented in Figure 7. Discriminating ability was the highest at Tyumen SAU (0.669), with three other sites falling behind by 42–62%. Chelyabinsk ARI best represented the whole target environment (value 7.388), followed by Kurgan Seeds (6.738, −10%), Omsk SAU (6.471, −14%), and Tyumen SAU (6.448, −15%).

These results are supported by the correlations between the yield at individual locations and the overall trial yield (Table 3). Germplasm performance for grain yield at Chelyabinsk ARI consistently had the highest correlation with the overall yield across four sites (0.85–0.89), while the lowest was at Kurgan Seeds and Omsk SAU. Discriminating ability was positively associated with the site yield: Tyumen SAU had the maximum grain yield among all sites and the highest discriminating ability. Representation ability is not directly related to the grain yield and discriminating ability. While Tyumen SAU had the highest yield and discriminating ability, it was the lowest for representation capacity. Chelyabinsk ARI, with the second highest yield, was the best at predicting performance across all sites.

### 3.3. Identification of Superior Germplasm for the Region

#### 3.3.1. Origin of High-Yielding Genotypes at Study Locations

KASIB trial comprised material from over 12 breeding programs, and it is logical to assume that locally bred cultivars and breeding lines would be more competitive in grain yield as compared to “outside” germplasm. This was rarely the case. For each KASIB trial, the average yield of genotypes across all four breeding stations was calculated and ranked based on two years of testing. The share of locally and regionally bred material in the top ten highest yielding performers is presented in Table S8. In all three KASIB trials, the material developed outside of the study regions dominated the group of best yield performers and accounted for over 60% of the top 10 yielders.

Locally developed genotypes did not make it into the highest productivity material in 6 cases out of 12. Germplasm from Omsk SAU performed relatively well at four study sites in 2019–20, meanwhile it was Kurgan Seeds in 2022–23 and Chelyabinsk ARI in 2023–24. High-yielding competitive germplasm in KASIB trials originated from as far away as Samara in the Volga region and other programs from Russia and Kazakhstan.

#### 3.3.2. Identification of Superior Germplasm Using GGE Analysis

GGE stability values (SV) measure the stability of genotypes across environments based on the first two principal components of the interaction effects. SV rank for genotypes combined with the yield rank defines the yield stability index (YSI). Five wheat genotypes with the best YSI in each trial along with the highest yielding check are presented in Table 4. Surprisingly, two breeding lines from Samara ARI are among the top five genotypes in all three trials. All spring wheat material from Samara demonstrated high leaf rust resistance, which probably contributed to its performance.

The line KS 111-09-2 from the Kurgan Seed company had the best YSI in the 2019–20 trial. Cultivars Kasibovskaya 2 from Omsk SAU and Pamyati Tyunina from Chelyabinsk ARI were among the best five in 2023–2024. There was limited variation among the best lines for the number of days to heading—they were within 2–3 days. The majority of the genotypes represented the tall stature material with a height close to or above 80 cm. Only two shorter lines were among the best material: Lutescens 8-12-18 and Line 155-A-1 from Kazakhstan. Substantial variation was observed for TKW, with two genotypes (KS 111-09-2 and Lut. 77-201-09) exceeding 40 g. A majority of the material presented in Table 4 was resistant to leaf rust under Chelyabinsk conditions. Superior cultivars and breeding lines identified in KASIB trials outperformed the local checks and represent valuable material as new varieties and parents for crosses.

## 4. Discussion

### 4.1. Climate Change in the Study Region and Its Effect on Spring Wheat

Climate change has become a factor defining many processes on Earth including agriculture. The International Panel on Climate Change (IPCC) [19] defines it as “a change in the state of the climate that can be identified (e.g., by using statistical tests) by changes in the mean and/or the variability of its properties and that persists for an extended period, typically decades or longer”. The IPCC uses a 30-year averaging period to distinguish climate change from short-term weather variability. The current study used 24 years of production and weather data, which is marginally suitable for use for a climate change approach. From a practical perspective, we find it more useful to refer to weather variation rather than “climate change”, which has been overused in the literature, frequently replacing the yearly and seasonal variation in key meteorological parameters.

Rosgidromet [10] published an increasing precipitation tendency in Western Siberia. The current study demonstrated that Ural Mountains–Western Siberia study sites were subjected to a gradual reduction in rainfall and increase in air temperature, especially in April. Analysis of the production yields showed a high positive yield dependence on rainfall in May, June, and April–August and a negative correlation with air temperature in May and the summer months.

The direction of weather variation induced by climatic change negatively affected spring wheat yields in this study. Several publications [11,20,21] anticipated a spring cereals yield increase due to climate change including the Siberian part of Russia. Perhaps larger models used by climate change studies provided a different picture compared to a precise focus on specific regions and crops. Despite the negative climate change, the yield gain tendency in spring wheat was demonstrated across all four study regions. This production gain can be attributed to technological advances, new cultivars, a higher use of inputs, and better machinery [22,23].

### 4.2. Yield Gap Between On-Station and On-Farm Yields

The main question of this study was how four spring wheat breeding programs in the study regions are positioned to develop competitive cultivars considering their ecology and using KASIB network trials as a tool for deciphering genotype–environment interactions. A biplot analysis used 24 years to group the environments based on similarities in the regional spring wheat yield, rainfall, and air temperature. According to results, Tyumen can be served by a breeding program targeting specific adaptation to the region. Omsk and Kurgan regions can be addressed by breeding programs developing germplasm with broader adaptation to these two regions. The resulting cultivars may also be adapted to Chelyabinsk conditions, which, in turn, require more specific adaptation to drier and warmer conditions.

Analysis of three biannual KASIB trials (2019–20, 2021–22, and 2023–24) at four breeding institutions demonstrated a substantial yield gap between research and production fields, higher than reported earlier [24]. The gap was the highest in Chelyabinsk at more than twice the higher yield difference and the lowest in Kurgan at 75%. The yearly yield variation between research stations and production fields did not match in Omsk and Tyumen. In fact, high production yields were associated with lower research yields resulting in negative correlations. The coincidence between production and research yields was best at Kurgan and Chelyabinsk. This misrepresentation of the target environments by the research programs is a concern, as germplasm developed at these institutions may not be sufficiently adapted to the broader production systems. The new enviromics approach, which integrates statistics, envirotyping (i.e., determining environmental factors), and remote sensing, could be used to unravel the complex interplay of genetics, environment, and management (Resende et al., 2024) [25]. Cooper et al.’s [26] review of the yield gap associated with breeding environments suggests designing crop improvement strategies that can explore genotype–management technology opportunities for the target population of environments and enable practical exploitation of the associated improved crop productivity under on-farm conditions.

### 4.3. Genotype–Environment Interaction and Evaluation of Breeding Sites

The breeding sites varied in the expression of two important adaptation traits: the rate of development evaluated by the number of days from emergence to heading and plant height. The more northern site, Tyumen, had faster development and taller plants, while Kurgan had a longer period to heading and shorter stature wheat. Importantly, the number of days to heading was positively correlated with the grain yield, especially in higher yielding environments. Plant height also showed a positive effect on grain yield in some sites and years. This association was previously reported in the region [6]. The variation in adaptation traits at different sites and years and its variable association with grain yield is the main cause for the genotype–environment interactions reported in this study.

GGE analysis ranked the breeding sites according to their suitability for germplasm field evaluation using three KASIB trials. Discriminating ability was the highest at Tyumen SAU, with three other sites falling behind. However, Chelyabinsk ARI best represented the whole target environment, followed by Kurgan Seeds, Omsk SAU, and Tyumen SAU. Discriminating ability was positively associated with the site yield, while representation ability was not directly related to yield or discriminating ability. GGE and similar types of analysis are widely used for the evaluation of genotype–environment interactions, with a primary focus on germplasm performance [27]. The paradigm of genotype–environment interactions has been evolving to add management practices [26], which is well justified for the study region. This subject still remains underexplored and deserves focused attention on spring wheat and other crops.

### 4.4. Competitiveness of Locally Developed Spring WHEAT Germplasm

Locally developed breeding materials are supposed to have a “home field advantage” [28]. The spring wheat germplasm performance in trials was disappointing, as domestic cultivars and breeding lines did not compete well with material from outside of the region. In all three KASIB trials, the material developed outside dominated in the group of best yield performers and accounted for over 60% of the top 10 yielders. The line KS 111-09-2 from the Kurgan Seed company had the best YSI in the 2019–20 trial. Cultivars Kasibovskaya 2 from Omsk SAU and Pamyati Tyunina from Chelyabinsk ARI were among the best five in 2023–2024. However, the best performing material originated from Samara ARI in the Volga region—almost 1000 km to the west of Chelyabinsk. Success of the spring wheat germplasm from Samara has been proven beyond the current study, as it performs well across all KASIB sites. It can partly be explained by its leaf rust resistance. Also, spring wheat breeding programs in the Volga region including Samara ARI have been united in the ECADA (Ecological Adaptation) program, which has a more integrated breeding framework including coordinated population exchanges and selections [29]. Perhaps this is one of the reasons for developing broadly adapted competitive germplasm. Spring durum wheat KASIB has also been dominated by the superior performance of Samara germplasm. It is certainly worth studying this subject to find the biological and genetic factors behind such a broad adaptation and superior yield performance.

Ural Mountains–Western Siberia region production system increasingly utilizes foreign cultivars, especially for hybrid crops. Shorter stature cultivars of spring wheat, like Granny, have been increasing their market share recently due to yield, lodging tolerance, and suitability for modern machinery [30]. Wheat is a cosmopolitan crop being developed in one area but may be grown widely on different continents, as demonstrated by CIMMYT germplasm [16]. A similar situation is in the Russia–Kazakhstan spring wheat belt, where more than a hundred formally released cultivars of diverse origin are available to producers. Local breeding programs ought to respond to the challenges and utilize relevant strategies and methodologies to stay competitive.

### 4.5. Perspectives

Spring wheat cultivars developed by the four breeding institutions included in this study still maintain a sizable market share in their respective regions and beyond. However, the current study also identified the challenges associated with the poor representation of target breeding environments. This can be amended by changing the agronomy and expansion of breeding and evaluation sites beyond the main experimental fields including on-farm trials.

Cooperation and integration between the institutions in developing competitive cultivars is very important for breeding success [31]. Currently, it is limited to KASIB trials and the occasional exchange of advanced material. It can certainly incorporate joint crossing and breeding programs to improve priority traits such as disease and drought resistance, grain quality, lodging tolerance, and others. Perhaps stronger linkages are needed with the breeding institutions, which contribute regionally competitive material like Samara ARI.

The study did not analyze the breeding methodology and use of modern genomic and phenomic tools. However, there is great potential in the development and use of molecular markers of genes controlling agronomic traits [6]. Spring wheat production in this important region is on the rise and producers require cultivars which fully satisfy their needs for adaptation, grain yield and quality, disease resistance, and suitability for their technologies. The breeding program ought to evolve to deliver such cultivars.

## 5. Conclusions

The study arrived at the following conclusions.

1. Climate change in the Ural Mountains–Western Siberia spring wheat production region observed during the period of 2001–2024 has a tendency toward the gradual reduction in precipitation and increase in air temperatures, especially in the month of April. These climatic changes are negatively correlated with spring wheat yields. The yield gains observed across four study regions (Chelyabinsk, Kurgan, Omsk, and Tyumen) during 2021–24 can be attributed to new, more responsive cultivars and technological and technical improvements.
2. Based on regional spring wheat yield, rainfall, and air temperature, the region was divided into subregions: Tyumen in the North has a quite distinct high grain yield; the Chelyabinsk region is characterized by lower precipitation and a lower grain yield; Omsk and Kurgan are similar in most years and are sometimes also similar to Tyumen and Chelyabinsk.
3. Environments at the four breeding programs (Chelyabinsk ARI, Kurgan Seeds, Omsk SAU, and Tyumen SAU) do not fully reflect the target production areas due to a very high yield gap and lack of association between the research and production yields at Omsk and Tyumen. This requires adjustment of the breeding evaluation framework to cover the main diversity of the target areas.
4. Genotype–environment interaction analysis showed that, among the breeding programs, Tyumen SAU has the highest yield and best discriminating ability while Chelyabinsk ARI best represents the whole target region. This is an important consideration for planning cooperative germplasm evaluation and joint research programs.
5. The majority of the highest yielding material in KASIB trials originated from outside of the region, proving the limited competitiveness of the local germplasm. Spring wheat breeding programs in the region ought to improve and evolve to maintain a competitive edge in the face of the increasing proliferation of foreign and Russian cultivars from other regions.

## Figures and Tables

**Table 1:** Geographical location and average weather parameters (2001–2024) for four spring wheat breeding programs.

Institution	Location Name	Latitude, North	Longitude, East	Precipitation, mm		Air Temperature, °C	
				Yearly	May–August	Yearly	May–August
Chelyabinsk ARI	Timiryasevo	54.936369	60.743553	382	188	3.8	18.0
Kurgan Seeds	Sadovoe	55.263211	65.095410	386	193	3.3	17.4
Omsk SAU	Omsk	55.025826	73.310101	427	212	2.6	17.0
Tyumen SAU	Tyumen	57.161225	65.337724	464	242	2.7	16.2

**Table 2:** Grain yield of KASIB trials and respective regions, adaptation, and agronomic traits, 2019–2024.

Site	Yield				Germination–Heading, Days	Plant Height, cm	1000 Kernel Weight, g
	KASIB		Region				
	t/ha	CV, %	t/ha	CV, %			
Chelyabinsk ARI	3.15	39.5	1.34	28.2	42.3	75.5	37.7
Kurgan Seed	2.92	52.0	1.61	24.7	44.9	69.3	33.1
Omsk SAU	2.72	33.9	1.52	13.0	41.7	79.3	39.2
Tyumen SAU	4.14	31.0	2.17	16.6	40.1	79.7	35.5
LSD 0.05	1.28	-	0.42	-	4.1	6.3	5.2

**Table 3:** Coefficients of correlations between genotypes tested in the same trial at different sites and years for grain yield (2019–24).

	Coefficients of Correlation Between the Average Yield Performance in KASIB Trials		
	2019–20	2021–22	2023–24
Chelyabinsk–Chelyabinsk	−0.19	0.36 *	0.25
Kurgan–Kurgan	0.47 ***	0.25	0.20
Omsk–Omsk	0.18	0.50 ***	0.10
Tyumen–Tyumen	0.28	0.38 *	0.02
Chelyabinsk–Kurgan	0.73 ***	0.50 ***	0.49 ***
Chelyabinsk–Omsk	0.57 **	0.46 ***	0.66 ***
Chelyabinsk–Tyumen	0.54 ***	0.58 ***	0.60 ***
Kurgan–Omsk	0.31 *	0.62 ***	0.35 *
Kurgan–Tyumen	0.46 ****	0.49 ***	0.33 *
Omsk–Tyumen	0.31 *	0.33 *	0.44 **
Chelyabinsk–Overall Mean	0.89 ***	0.85 ***	0.86 ***
Kurgan–Overall Mean	0.76 ***	0.80 ***	0.66 ***
Omsk–Overall Mean	0.74 ***	0.72 ***	0.74 ***
Tyumen–Overall Mean	0.73 ***	0.81 ***	0.84 ***

*, ** and ***—statistically significant at *p* > 0.05; 0.01 and 0.001, respectively.

**Table 4:** Agronomic traits of superior genotypes with high yield stability index (YSI) identified in three KASIB trials, 2019–24.

Entry #	Variety	Originator	Yield, kg/ha	Yield Rank	YSI	Days to Heading	Plant Height, cm	TKW, g
KASIB 2019–20								
25	Saratovskaya 29	Historical check	3.00	35	40	41.3	81.4	36.8
32	KS 111-09-2	Kurgan Seed	3.45	5	14	44.1	79.7	40.2
47	L396 (Favorit)	Saratov ARI	3.44	6	16	43.5	81.0	34.9
33	Line-1616ae14	Samara ARI	3.70	1	17	44.6	81.0	37.8
35	Line-1617ae9	Samara ARI	3.57	3	20	43.8	81.3	37.1
28	Lutescens TP-64	Kurgan ARI	3.31	15	21	43.1	78.8	32.1
KASIB 2021–22								
20	Tertsiya	Local check	3.42	29	43	39.5	83.5	36.8
32	Lutescens 1486	Samara ARI	3.97	6	11	41.9	78.2	35.4
33	Lutescens 1489	Samara ARI	3.98	5	13	41.5	87.0	35.0
8	Lut. 77-201-09	Karabalyk AES	3.71	12	16	43.1	88.0	40.4
26	Line Pt-311	Kurgan ARI	3.66	14	16	40.1	85.4	36.6
10	Lutescens 8-12-18	Karabalyk AES	3.61	16	22	39.6	70.2	36.4
KASIB 2023–24								
25	Astana 2	Local check	3.02	18	42	43.8	74.9	33.2
35	Lutescens 1535	Samara ARI	3.43	4	6	46.9	73.7	35.1
40	Kasibovskaya 2	Omsk SAU	3.32	6	10	45.3	74.1	36.5
18	Line 155-A-1	E.-Kazakhstan EF	3.26	9	17	45.5	61.7	32.8
45	Pamyati Tyunina	Chelyabinsk ARI	3.52	2	17	42.4	73.7	32.0
48	Line 1616ae14	Samara ARI	2.88	25	26	44.1	65.4	29.9

## Data Availability

Regional weather and crop productivity data is available in the open sources mentioned in the paper. KASIB trials data availability is a subject to the network rules and regulations.

## Supplementary Materials

The following supporting information can be downloaded at: https://www.mdpi.com/article/10.3390/plants14213256/s1; Table S1: Spring wheat germplasm included in KASIB trials; Table S2: Monthly, yearly and seasonal (May–August) precipitation for three 8-years periods from 2001 till 2024 in four study regions; Table S3: Monthly, yearly and seasonal (May–August) air temperature for three 8-years periods from 2001 till 2024 in four study regions; Table S4: Coefficients of correlation between spring wheat grain yield and weather parameters during the growing season in four regions in 2001–2024; Table S5: Grain yield of KASIB trials and respective regions, adaptation and agronomic traits, 2019–2024; Table S6: Coefficients of correlation between adaptation traits, 1000 KW and grain yield in three KASIB trials, 2019–24; Table S7: ANOVA results for grain yield; and Table S8: Origin of high-yielding genotypes in KASIB trials at four breeding programs, 2019–24.

## Abbreviations

The following abbreviations are used in this manuscript:

ARI	Agricultural Research Institute
ASV	AMMI stability value
DH	Number of days from emergence to heading
GGE	Genotype—environment interaction
IPCC	International Panel on Climate Change
KASIB	Kazakhstan-Siberia Network on Spring Wheat Improvement
PH	Plant height
SAU	State Agrarian University
TKW	1000 kernel weight
YSI	Yield stability index
