# Supplementary material for: Climate, Weather, and Ecology in Evaluation of High Latitude Spring Wheat Breeding Sites and Germplasm
Source: Plants (Basel). 2025 Oct 24;14(21):3256. doi: 10.3390/plants14213256 (PMC12608430; doi:10.3390/plants14213256)
Supplement: Supplementary file 1 [file plants-14-03256-s001.zip › plants-3933800-supplementary.pdf]

**Table S1.** Spring wheat germplasm included in KASIB trials.

| Nursery | # plot | Variety | Country | Originator |
|---|---|---|---|---|
| 19-20 KASIB | 1 | Line-R-1415 | KAZ | Aktobe ARS |
| 19-20 KASIB | 2 | Line-R-1417 | KAZ | Aktobe ARS |
| 19-20 KASIB | 3 | Stepnaya 150 | KAZ | Aktobe ARS |
| 19-20 KASIB | 4 | GVK 2097/14 | KAZ | East-Kazakhstan ARI |
| 19-20 KASIB | 5 | GVK 2140/6 | KAZ | East-Kazakhstan ARI |
| 19-20 KASIB | 6 | Lutescens 762 | KAZ | Kazakh ARI |
| 19-20 KASIB | 7 | Lutescens 799 | KAZ | Kazakh ARI |
| 19-20 KASIB | 8 | Taymas | KAZ | Shortandy ARI |
| 19-20 KASIB | 9 | Line-67/98-13 | KAZ | Shortandy ARI |
| 19-20 KASIB | 10 | Lutescens 111/09 | KAZ | Karabalyk ARS |
| 19-20 KASIB | 11 | Stepnodar 90 | KAZ | Karabalyk ARS |
| 19-20 KASIB | 12 | Erithrospermum 79/07 | KAZ | Karabalyk ARS |
| 19-20 KASIB | 13 | Lutescens 1991 | KAZ | Karaganda ARI |
| 19-20 KASIB | 14 | Lutescens 2055 | KAZ | Karaganda ARI |
| 19-20 KASIB | 15 | Lutescens 2174 | KAZ | Karaganda ARI |
| 19-20 KASIB | 16 | Line- 11/09-13-3 | KAZ | Pavlodar ARI |
| 19-20 KASIB | 17 | Line- 37/07-12-2 | KAZ | Pavlodar ARI |
| 19-20 KASIB | 21 | Pamyaty Aziyeva | RUS | Early Ecological check |
| 19-20 KASIB | 22 | Tertsiya | RUS | LR resistant Ecological check |
| 19-20 KASIB | 23 | Astana 2 | KAZ | Medium Ecological check |
| 19-20 KASIB | 24 | Omskaya 35 | RUS | Late Ecological check |
| 19-20 KASIB | 25 | Saratovskaya 29 | RUS | Historical Ecolocical check |
| 19-20 KASIB | 26 | Lider 80 | RUS | Barnaul ARI |
| 19-20 KASIB | 27 | Lutescens 1143 | RUS | Barnaul ARI |
| 19-20 KASIB | 28 | Lutescens TP-64 | RUS | Kurgan ARI |
| 19-20 KASIB | 29 | Lutescenss ST-335 | RUS | Kurgan ARI |
| 19-20 KASIB | 30 | KS 115/09-1 | RUS | Kurganseed |
| 19-20 KASIB | 31 | KS 161/08-2p | RUS | Kurganseed |
| 19-20 KASIB | 32 | KS 111/09-2 | RUS | Kurganseed |
| 19-20 KASIB | 33 | Line- 1616ae14 | RUS | Samara ARI |
| 19-20 KASIB | 34 | Line- 1643ae3 | RUS | Samara ARI |
| 19-20 KASIB | 35 | Line- 1617ae9 | RUS | Samara ARI |
| 19-20 KASIB | 36 | Line- 2026 | RUS | Novosibirsk ARI |
| 19-20 KASIB | 37 | Line- 2149 | RUS | Novosibirsk ARI |
| 19-20 KASIB | 38 | Lutescens 123-13 | RUS | Omsk SAU |
| 19-20 KASIB | 39 | Silantiy | RUS | Omsk SAU |
| 19-20 KASIB | 40 | Lutescens 128-15 | RUS | Omsk SAU |
| 19-20 KASIB | 41 | Lutescens 13-15 | RUS | Omsk SAU |
| 19-20 KASIB | 42 | Lutescens 417/10-5 | RUS | Omsk ARI |
| 19-20 KASIB | 43 | L70/06-4 | RUS | Omsk ARI |
| 19-20 KASIB | 44 | L14/10-14 | RUS | Omsk ARI |
| 19-20 KASIB | 45 | GAU 21-2018 | RUS | SAU Tyumen |
| 19-20 KASIB | 46 | GAU 6-2018 | RUS | SAU Tyumen |

| | | | | |
|---|---|---|---|---|
| 19-20 KASIB | 47 | L396=Favorit | RUS | Saratov ARI |
| 19-20 KASIB | 48 | Lutescens 375 | RUS | Saratov ARI |
| 19-20 KASIB | 49 | Erithrospermum 25787 | RUS | Chelyabinsk ARI |
| 19-20 KASIB | 50 | Chelyaba 80 | RUS | Chelyabinsk ARI |
| 19-20 KASIB | 51 | Ilmenskaya 2 | RUS | Chelyabinsk ARI |
| 19-20 KASIB | 52 | Orenurgskaya 22 | RUS | Orenburg ARI |
| 19-20 KASIB | 53 | Orenburgskaya 23 | RUS | Orenburg ARI |
| 19-20 KASIB | 54 | Orenurgskaya yubil. | RUS | Orenburg ARI |
| 19-20 KASIB | 55 | Silach | RUS | Chelyabinsk ARI |
| | | | | |
| 21-22 KASIB | 1 | Dinastiya | KAZ | Aktobe ARS |
| 21-22 KASIB | 2 | Line 198/225-2020 | KAZ | Aktobe ARS |
| 21-22 KASIB | 3 | Line 205-2020 | KAZ | Aktobe ARS |
| 21-22 KASIB | 4 | Lutescens 176/09 | KAZ | Shortandy ARI |
| 21-22 KASIB | 5 | Lutescens 342/08 | KAZ | Shortandy ARI |
| 21-22 KASIB | 6 | Line 43/94к-07-7 | KAZ | Pavlodar ARI |
| 21-22 KASIB | 7 | Line 2/03-09-3 | KAZ | Pavlodar ARI |
| 21-22 KASIB | 8 | Lutescens 77  201/09 | KAZ | Karabalyk ARS |
| 21-22 KASIB | 9 | Lutescens 30 22/09 | KAZ | Karabalyk ARS |
| 21-22 KASIB | 10 | Lutescens 8-12-18 | KAZ | Karabalyk ARS |
| 21-22 KASIB | 11 | Lutescens 2244 | KAZ | Karaganda ARI |
| 21-22 KASIB | 12 | Lutescens 2219 | KAZ | Karaganda ARI |
| 21-22 KASIB | 13 | Lutescens 2223 | KAZ | Karaganda ARI |
| 21-22 KASIB | 14 | Line 23/07 | KAZ | North Kazakh ARI |
| 21-22 KASIB | 15 | Line 435/12 | KAZ | North Kazakh ARI |
| 21-22 KASIB | 19 | Pamyaty Aziyeva | RUS | Early Ecological check |
| 21-22 KASIB | 20 | Tertsiya | RUS | LR resistant Ecological check |
| 21-22 KASIB | 21 | Astana 2 | KAZ | Medium Ecological check |
| 21-22 KASIB | 22 | Omskaya 35 | RUS | Late Ecological check |
| 21-22 KASIB | 23 | Saratovskaya 29 | RUS | Historical Ecolocical check |
| 21-22 KASIB | 24 | Line Chtr-11 | RUS | Kurgan ARI |
| 21-22 KASIB | 25 | Line Pt-235 | RUS | Kurgan ARI |
| 21-22 KASIB | 26 | Line Pt-311 | RUS | Kurgan ARI |
| 21-22 KASIB | 27 | KS 14/09-2 | RUS | Kurgan Seeds |
| 21-22 KASIB | 28 | KS 60/09-9 | RUS | Kurgan Seeds |
| 21-22 KASIB | 29 | KS 61/09-4 | RUS | Kurgan Seeds |
| 21-22 KASIB | 30 | KS 285/12-1586 | RUS | Kurgan Seeds |
| 21-22 KASIB | 31 | Lutescens 1462 | RUS | Samara ARI |
| 21-22 KASIB | 32 | Lutescens 1486 | RUS | Samara ARI |
| 21-22 KASIB | 33 | Lutescens 1489 | RUS | Samara ARI |
| 21-22 KASIB | 34 | Lutescens 1356 | RUS | Novosibirsk  ARI |
| 21-22 KASIB | 35 | Lutescens 1364 | RUS | Novosibirsk  ARI |
| 21-22 KASIB | 36 | Agronomicheskaya 5 | RUS | Omsk SAU |
| 21-22 KASIB | 37 | Lutescens 76-17 | RUS | Omsk SAU |
| 21-22 KASIB | 38 | Lutescens.82/09-7 | RUS | Omsk ARI |
| 21-22 KASIB | 39 | Lutescens136/10-1 | RUS | Omsk ARI |

| | | | | |
|---|---|---|---|---|
| 21-22 KASIB | 40 | Lutescens71/10-4 | RUS | Omsk ARI |
| 21-22 KASIB | 41 | Yalutorovka | RUS | SAU Tyumen |
| 21-22 KASIB | 42 | GAU-11-2016 | RUS | SAU Tyumen |
| 21-22 KASIB | 43 | Chelyabinka | RUS | Chelyabinsk ARI |
| 21-22 KASIB | 44 | Line1616ae14 | RUS | Samara ARI |
| | | | | |
| 23-24 KASIB | 1 | Line 201м/22 | KAZ | Aktobe ARS |
| 23-24 KASIB | 2 | Line 334м/22 | KAZ | Aktobe ARS |
| 23-24 KASIB | 3 | Line 337м/22 | KAZ | Aktobe ARS |
| 23-24 KASIB | 4 | Line 55/08 | KAZ | Shortandy ARI |
| 23-24 KASIB | 5 | Line 143/09 | KAZ | Shortandy ARI |
| 23-24 KASIB | 6 | Line 42/93-09-1 | KAZ | Pavlodar ARI |
| 23-24 KASIB | 7 | Line 1205-09-8 | KAZ | Pavlodar ARI |
| 23-24 KASIB | 8 | Lutescens 54 190/09 | KAZ | Karabalyk ARS |
| 23-24 KASIB | 9 | Lutescens 20 161/08 | KAZ | Karabalyk ARS |
| 23-24 KASIB | 10 | Kudesnitsa | KAZ | Karabalyk ARS |
| 23-24 KASIB | 11 | Lutescens 2216 | KAZ | Karaganda ARI |
| 23-24 KASIB | 12 | Lutescens 2222 | KAZ | Karaganda ARI |
| 23-24 KASIB | 13 | Sary Arka 27 | KAZ | Karaganda ARI |
| 23-24 KASIB | 14 | Line 218/10 | KAZ | North Kazakh ARI |
| 23-24 KASIB | 15 | PSI b 12 I 453 | KAZ | North Kazakh ARI |
| 23-24 KASIB | 16 | PSI b 12 I I 189 | KAZ | North Kazakh ARI |
| 23-24 KASIB | 17 | Line 98-A-2 | KAZ | East-Kazakhstan OPXMK |
| 23-24 KASIB | 18 | Line 155-A-1 | KAZ | East-Kazakhstan OPXMK |
| 23-24 KASIB | 19 | Line 249-A-25 | KAZ | East-Kazakhstan OPXMK |
| 23-24 KASIB | 23 | Pamyaty Aziyeva | RUS | Early Ecological check |
| 23-24 KASIB | 24 | Tertsiya | RUS | LR resistant Ecological check |
| 23-24 KASIB | 25 | Astana 2 | KAZ | Medium Ecological check |
| 23-24 KASIB | 26 | Omskaya 35 | RUS | Late Ecological check |
| 23-24 KASIB | 27 | Saratovskaya 29 | RUS | Historical Ecolocical check |
| 23-24 KASIB | 28 | L-407/ChT | RUS | Kurgan ARI |
| 23-24 KASIB | 29 | L-6/CM | RUS | Kurgan ARI |
| 23-24 KASIB | 30 | L-235/PT | RUS | Kurgan ARI |
| 23-24 KASIB | 31 | KS 39/08-7 | RUS | Kurgan Seeds |
| 23-24 KASIB | 32 | KS 29/17u | RUS | Kurgan Seeds |
| 23-24 KASIB | 33 | Lutescens 1485 | RUS | Samara ARI |
| 23-24 KASIB | 34 | Lutescens 1510 | RUS | Samara ARI |
| 23-24 KASIB | 35 | Lutescens 1535 | RUS | Samara ARI |
| 23-24 KASIB | 36 | L373 | RUS | Saratov ARI |
| 23-24 KASIB | 37 | L447 | RUS | Saratov ARI |
| 23-24 KASIB | 38 | L2203 | RUS | Novosibirsk ARI |
| 23-24 KASIB | 39 | L1353 | RUS | Novosibirsk ARI |
| 23-24 KASIB | 40 | Kasibovskaya 2 | RUS | Omsk SAU |
| 23-24 KASIB | 41 | Lutescens 34-16 | RUS | Omsk SAU |
| 23-24 KASIB | 42 | Lutescens.205/12-5 | RUS | Omsk ARI |
| 23-24 KASIB | 43 | Lutescens.242/13-10 | RUS | Omsk ARI |

| | | | | |
|---|---|---|---|---|
| 23-24 KASIB | 44 | Lutescens 74/16-1 | RUS | Omsk ARI |
| 23-24 KASIB | 45 | Pamyati Tyunina | RUS | Chelyabinsk ARI |
| 23-24 KASIB | 46 | Zagadka | RUS | Chelyabinsk ARI |
| 23-24 KASIB | 47 | Erithrospermum 26464 | RUS | Chelyabinsk ARI |
| 23-24 KASIB | 48 | Line1616ae 14 | RUS | Samara ARI |

**Table S2.** Monthly, yearly and seasonal (May-August) precipitation for three 8-years periods from 2001 till 2024 in four study regions.

| Region | Years | Prec., mm 1 | Prec., mm 2 | Prec., mm 3 | Prec., mm 4 | Prec., mm 5 | Prec., mm 6 | Prec., mm 7 | Prec., mm 8 | Prec., mm 9 | Prec., mm 10 | Prec., mm 11 | Prec., mm 12 | Prec., mm Year | Prec., mm May-August | Region Yield, t/ha |
|---|---|---|---|---|---|---|---|---|---|---|---|---|---|---|---|---|
| Tyumen | 01-08 | 21.7 | 17.7 | 27.0 | 17.0 | 49.4 | 75.1 | 97.4 | 60.8 | 44.8 | 42.3 | 36.9 | 20.3 | 510.5 | 282.6 | 2.00 |
| | 09-16 | 22.0 | 12.3 | 26.5 | 34.5 | 45.9 | 50.5 | 100.6 | 38.2 | 36.0 | 53.9 | 30.3 | 30.1 | 480.7 | 235.1 | 1.84 |
| | 17-24 | 21.5 | 21.3 | 23.4 | 18.3 | 46.9 | 72.4 | 57.8 | 59.2 | 30.4 | 31.8 | 30.0 | 28.3 | 441.2 | 236.2 | 2.15 |
| | *01-24* | *20.1* | *16.9* | *25.4* | *23.3* | *44.1* | *68.0* | *76.7* | *53.2* | *38.7* | *37.4* | *35.8* | *25.1* | *464.5* | *241.9* | *20.3* |
| Kurgan | 01-08 | 17.5 | 16.5 | 21.6 | 18.0 | 53.8 | 61.7 | 46.6 | 50.8 | 42.0 | 34.5 | 24.0 | 15.4 | 402.3 | 212.9 | 1.42 |
| | 09-16 | 22.8 | 13.1 | 22.1 | 29.7 | 33.0 | 28.2 | 105.2 | 46.5 | 25.3 | 49.6 | 36.2 | 28.5 | 440.1 | 212.9 | 1.45 |
| | 17-24 | 21.6 | 19.4 | 21.1 | 21.8 | 36.6 | 43.8 | 50.0 | 57.3 | 24.6 | 33.6 | 25.6 | 20.1 | 375.4 | 187.6 | 1.57 |
| | *01-24* | *18.8* | *15.4* | *21.4* | *22.0* | *38.2* | *49.1* | *57.0* | *48.6* | *30.8* | *35.7* | *28.7* | *20.3* | *386.0* | *192.9* | *15.0* |
| Omsk | 01-08 | 23.0 | 18.9 | 22.3 | 23.4 | 29.6 | 77.8 | 69.7 | 55.5 | 28.6 | 30.8 | 39.9 | 24.6 | 443.9 | 232.6 | 1.43 |
| | 09-16 | 26.3 | 16.1 | 27.5 | 37.1 | 28.5 | 45.7 | 79.2 | 47.1 | 24.2 | 59.2 | 36.0 | 37.6 | 464.5 | 200.5 | 1.49 |
| | 17-24 | 21.4 | 19.5 | 21.7 | 22.0 | 35.0 | 51.1 | 67.3 | 41.9 | 33.4 | 32.4 | 30.8 | 25.3 | 401.7 | 195.2 | 1.52 |
| | *01-24* | *21.1* | *17.9* | *22.3* | *26.0* | *31.5* | *58.3* | *70.1* | *51.9* | *28.2* | *35.4* | *37.0* | *27.0* | *426.7* | *211.9* | *14.8* |
| Chelyabinsk | 01-08 | 16.7 | 20.9 | 24.9 | 31.3 | 34.8 | 48.9 | 50.4 | 41.2 | 28.9 | 38.1 | 22.7 | 15.6 | 374.3 | 175.3 | 1.23 |
| | 09-16 | 26.2 | 19.8 | 25.7 | 29.6 | 43.7 | 45.2 | 77.0 | 63.7 | 26.3 | 32.5 | 24.0 | 23.7 | 437.4 | 229.6 | 1.42 |
| | 17-24 | 20.6 | 24.5 | 17.6 | 22.9 | 35.7 | 41.0 | 54.5 | 47.4 | 33.6 | 31.0 | 25.4 | 24.1 | 378.1 | 178.5 | 1.38 |
| | *01-24* | *19.5* | *20.0* | *22.7* | *25.3* | *35.4* | *44.8* | *58.4* | *49.7* | *28.3* | *32.3* | *24.3* | *21.1* | *381.9* | *188.3* | *12.8* |

**Table S3.** Monthly, yearly and seasonal (May-August) air temperature for three 8-years periods from 2001 till 2024 in four study regions.

| Region | Years | t, oC 1 | t, oC 2 | t, oC 3 | t, oC 4 | t, oC 5 | t, oC 6 | t, oC 7 | t, oC 8 | t, oC 9 | t, oC 10 | t, oC 11 | t, oC 12 | t, oC Year | t, oC May-August |
|---|---|---|---|---|---|---|---|---|---|---|---|---|---|---|---|
| Tyumen | 01-08 | -14.35 | -13.30 | -4.08 | 3.35 | 12.65 | 16.38 | 18.76 | 16.03 | 10.21 | 3.24 | -4.65 | -12.01 | 2.69 | 15.95 |
| | 09-16 | -16.30 | -10.15 | -4.60 | 5.35 | 12.08 | 17.78 | 17.50 | 17.38 | 10.18 | 0.35 | -6.70 | -11.45 | 2.62 | 16.18 |
| | 17-24 | -14.65 | -11.98 | -3.66 | 5.28 | 12.39 | 16.33 | 19.89 | 17.03 | 10.25 | 3.76 | -5.43 | -11.35 | 3.15 | 16.41 |
| | *01-24* | *-15.4* | *-12.9* | *-4.3* | *4.7* | *12.4* | *16.8* | *18.9* | *16.7* | *10.4* | *3.1* | *-5.5* | *-12.5* | *2.7* | *16.2* |
| Kurgan | 01-08 | -14.56 | -14.09 | -4.56 | 4.14 | 14.14 | 17.71 | 19.68 | 17.55 | 11.53 | 4.14 | -4.01 | -12.01 | 3.30 | 17.27 |
| | 09-16 | -16.55 | -11.45 | -4.88 | 6.00 | 13.33 | 18.98 | 18.10 | 18.03 | 10.93 | 1.55 | -6.03 | -10.93 | 3.09 | 17.11 |
| | 17-24 | -14.84 | -12.39 | -4.40 | 6.26 | 13.70 | 17.64 | 20.81 | 18.14 | 11.28 | 4.50 | -4.88 | -11.65 | 3.68 | 17.57 |
| | *01-24* | *-15.7* | *-13.7* | *-4.8* | *5.6* | *13.8* | *18.1* | *19.9* | *17.9* | *11.6* | *4.0* | *-4.8* | *-12.5* | *3.3* | *17.4* |
| Omsk | 01-08 | -16.28 | -14.38 | -5.43 | 3.44 | 14.03 | 18.15 | 19.10 | 16.73 | 11.14 | 3.89 | -4.80 | -13.38 | 2.68 | 17.00 |
| | 09-16 | -17.55 | -12.98 | -5.20 | 6.03 | 12.38 | 18.33 | 18.40 | 17.68 | 10.60 | 0.83 | -7.38 | -10.95 | 2.51 | 16.69 |
| | 17-24 | -15.56 | -14.25 | -5.49 | 5.61 | 13.29 | 17.70 | 20.34 | 17.65 | 10.90 | 4.40 | -5.94 | -13.10 | 2.96 | 17.24 |
| | *01-24* | *-17.0* | *-14.9* | *-5.6* | *5.1* | *13.2* | *18.1* | *19.4* | *17.2* | *11.1* | *3.7* | *-5.8* | *-13.8* | *2.6* | *17.0* |
| Chelyabinsk | 01-08 | -13.43 | -13.25 | -4.49 | 4.99 | 14.18 | 17.71 | 20.08 | 18.43 | 12.48 | 4.58 | -3.60 | -11.89 | 3.81 | 17.60 |
| | 09-16 | -15.18 | -11.48 | -4.78 | 5.83 | 14.48 | 19.78 | 18.73 | 19.10 | 12.08 | 2.35 | -4.63 | -10.05 | 3.85 | 18.02 |
| | 17-24 | -14.49 | -12.75 | -5.18 | 6.74 | 14.13 | 18.10 | 21.25 | 18.83 | 11.71 | 4.79 | -4.53 | -11.58 | 3.92 | 18.08 |
| | *01-24* | *-14.8* | *-13.5* | *-5.1* | *6.0* | *14.3* | *18.6* | *20.4* | *18.8* | *12.3* | *4.4* | *-4.1* | *-12.0* | *3.8* | *18.0* |

**Table S4.** Coefficients of correlation between spring wheat grain yield and weather parameters during the growing season in four regions in 2001-2024.

| **Months** | **Coefficients of correlation between grain yield and weather parameters:** | | | |
|---|---|---|---|---|
| | **Tyumen** | **Kurgan** | **Omsk** | **Chelyabinsk** |
| Rainfall | | | | |
| May | 0.24 | 0.08 | 0.25 | 0.36 |
| June | 0.55** | 0.45* | -0.07 | 0.54** |
| July | -0.11 | 0.31 | 0.56** | 0.23 |
| August | 0.12 | 0.11 | 0.33 | 0.29 |
| May-August | 0.33 | 0.50** | 0.47* | 0.70*** |
| Air temperature | | | | |
| May | -0.29 | -0.46* | -0.05 | -0.54** |
| June | -0.28 | -0.19 | -0.17 | -0.10 |
| July | -0.04 | -0.09 | -0.48* | -0.35 |
| August | -0.27 | -0.30 | -0.15 | -0.25 |
| May-August | -0.40* | -0.47* | -0.38 | -0.51* |

**Table S5.** Grain yield of KASIB trials and respective regions, adaptation and agronomic traits, 2019-2024.

| Year | Site | Yield, kg/ha | Germination-Heading, days | Plant height, cm | M1000 |
|---|---|---|---|---|---|
| 2019 | Chelyabinsk ARI | 3457 | 42.4 | 76.4 | 39.8 |
| 2020 | Chelyabinsk ARI | 868 | 45.0 | 48.5 | 33.0 |
| 2019-20 | Chelyabinsk ARI | 2162 | 43.7 | 62.4 | 36.4 |
| 2021 | Chelyabinsk ARI | 3775 | 36.5 | 75.9 | 39.0 |
| 2022 | Chelyabinsk ARI | 4408 | 40.6 | 87.7 | 39.9 |
| 2021-22 | Chelyabinsk ARI | 4092 | 38.6 | 81.8 | 39.4 |
| 2023 | Chelyabinsk ARI | 3721 | 44.3 | 73.7 | - |
| 2024 | Chelyabinsk ARI | 2725 | 47.0 | 97.2 | 36.2 |
| 2023-24 | Chelyabinsk ARI | 3223 | 45.6 | 85.4 | 36.2 |
| 2019-24 | Chelyabinsk ARI | 3151 | 42.3 | 75.5 | 37.7 |
| 2019 | Kurgan Seed | 3616 | 43.8 | 82.5 | 34.2 |
| 2020 | Kurgan Seed | 1918 | 45.4 | 79.5 | 31.2 |
| 2019-20 | Kurgan Seed | 2767 | 44.6 | 81.0 | 32.7 |
| 2021 | Kurgan Seed | 1818 | 39.4 | 42.5 | 34.6 |
| 2022 | Kurgan Seed | 5313 | 46.9 | 76.5 | 35.9 |
| 2021-22 | Kurgan Seed | 3565 | 43.1 | 59.5 | 35.3 |
| 2023 | Kurgan Seed | 1350 | 47.3 | 51.9 | 32.5 |
| 2024 | Kurgan Seed | 3005 | 48.6 | 81.0 | 28.2 |
| 2023-24 | Kurgan Seed | 2178 | 47.9 | 66.4 | 30.4 |
| 2019-24 | Kurgan Seed | 2919 | 44.9 | 69.3 | 33.1 |
| 2019 | Omsk SAU | 3467 | 45.0 | 103.1 | 42.6 |
| 2020 | Omsk SAU | 2657 | 40.8 | 83.1 | 45.3 |
| 2019-20 | Omsk SAU | 3062 | 42.9 | 93.1 | 44.0 |
| 2021 | Omsk SAU | 2430 | 40.8 | 82.2 | 37.2 |
| 2022 | Omsk SAU | 3208 | 41.0 | 82.9 | 39.8 |
| 2021-22 | Omsk SAU | 2819 | 40.9 | 82.6 | 38.5 |
| 2023 | Omsk SAU | 2345 | 43.0 | 68.0 | 33.9 |
| 2024 | Omsk SAU | 1796 | 39.2 | 39.2 | 32.5 |
| 2023-24 | Omsk SAU | 2071 | 41.1 | 53.6 | 33.2 |
| 2019-24 | Omsk SAU | 2723 | 41.7 | 79.3 | 39.2 |
| 2019 | Tyumen SAU | 4767 | 43.7 | 96.3 | 39.5 |
| 2020 | Tyumen SAU | 3918 | 36.4 | 88.8 | 35.6 |
| 2019-20 | Tyumen SAU | 4343 | 40.1 | 92.6 | 37.5 |
| 2021 | Tyumen SAU | 2051 | 34.2 | 49.9 | 32.7 |
| 2022 | Tyumen SAU | 5673 | 42.4 | 90.8 | 39.1 |
| 2021-22 | Tyumen SAU | 3862 | 38.3 | 70.4 | 35.9 |
| 2023 | Tyumen SAU | 5042 | 40.1 | - | 34.5 |

| | | | | | |
|---|---|---|---|---|---|
| 2024 | Tyumen SAU | 3538 | 43.6 | 81.0 | 32.9 |
| 2023-24 | Tyumen SAU | 4290 | 41.8 | 81.0 | 33.7 |
| 2019-24 | Tyumen SAU | 4143 | 40.1 | 79.7 | 35.5 |

**Table S6.** Coefficients of correlation between adaptation traits, 1000KW and grain yield in three KASIB trials, 2019-24.

| Site | Year | Difference (%) between the yearly and long-term values for: | | Yield, t/ha | Coefficients of correlation between grain yield and: | | |
|---|---|---|---|---|---|---|---|
| | | precipitation May-August | air temperature May-August | | Days to heading | Plant height | 1000 kernel weight |
| Chelyabinsk | 2019 | -13.4 | 0.7 | 3.46 | 0.26 | 0.38 | 0.38 |
| | 2020 | -29.5 | 6.9 | 0.87 | -0.33 | -0.77** | 0.38 |
| | 2021 | -51.4 | 14.4 | 3.77 | 0.47* | 0.60*** | 0.40* |
| | 2022 | -20.3 | -2.8 | 4.41 | 0.23 | -0.07 | 0.10 |
| | 2023 | 22.2 | 3.6 | 3.72 | 0.19 | 0 | - |
| | 2024 | 45.4 | -7.5 | 2.72 | 0.17 | -0.12 | 0.71*** |
| Kurgan | 2019 | 5.6 | -0.3 | 3.62 | 0.12 | 0.25 | 0.17 |
| | 2020 | -43.2 | 6.2 | 1.92 | 0.07 | 0.45* | 0.12 |
| | 2021 | -31.7 | 12.1 | 1.82 | 0.27 | 0.34 | 0.27 |
| | 2022 | -25.0 | -0.1 | 5.31 | 0.48* | -0.08 | 0.57** |
| | 2023 | -3.1 | 6.8 | 1.35 | 0.24 | 0.25 | 0.26 |
| | 2024 | 71.7 | -4.5 | 3.01 | 0.22 | 0.04 | 0.61 |
| Omsk | 2019 | -10.5 | -2.8 | 3.47 | -0.01 | 0.06 | 0.23 |
| | 2020 | -38.6 | 8.7 | 2.66 | 0.31 | 0.32 | 0.17 |
| | 2021 | -37.0 | 8.8 | 2.43 | 0.13 | - | - |
| | 2022 | 2.7 | 1.9 | 3.21 | 0.09 | - | - |
| | 2023 | -15.4 | 5.0 | 2.35 | 0.30 | -0.02 | 0.18 |
| | 2024 | 55.4 | -2.2 | 1.80 | 0.21 | 0.21 | 0.32 |
| Tyumen | 2019 | 22.4 | -2.3 | 2.05 | -0.12 | 0.41* | 0.33 |
| | 2020 | -21.6 | 6.9 | 5.67 | 0.41* | 0.42* | 0.06 |
| | 2021 | -60.1 | 13.7 | 5.04 | 0.26 | 0.34 | 0.27 |
| | 2022 | 12.5 | 1.4 | 3.54 | -0.15 | -0.27 | 0.14 |
| | 2023 | -33.4 | 8.2 | 4.77 | -0.01 | - | 0.30 |
| | 2024 | 18.3 | -4.8 | 3.92 | 0.02 | 0.14 | 0.57** |

*, ** and *** - statistically significant at P>0.05; 0.01 and 0.001, respectively.

**Table S7.** ANOVA results for grain yield.

| KASIB trial | Significance of main effects and interactions in KASIB trials: | | | | |
|---|---|---|---|---|---|
| | Site | Year | Genotypes | Year - sites | Genotypes - environments |
| 2019-20 | < 0.001 | < 0.001 | < 0.05 | < 0.01 | n.s. |
| 2021-22 | < 0.001 | < 0.001 | n.s. | < 0.01 | n.s. |
| 2023-24 | < 0.001 | < 0.001 | n.s. | < 0.01 | n.s. |

**Table S8.** Origin of high-yielding genotypes in KASIB trials at four breeding programs, 2019-24.

| **KASIB year** | **Evaluation site** | **Number of genotypes in top 10 highest yielding entries originating from:** | | | | |
|---|---|---|---|---|---|---|
| | | **Chelyabinsk ARI** | **Kurgan Seeds** | **Omsk SAU** | **Tyumen SAU** | **Others** |
| 2019-20 | Chelyabinsk ARI | 1 | 1 | 1 | 0 | 7 |
| | Kurgan Seeds | 0 | 0 | 2 | 1 | 7 |
| | Omsk SAU | 1 | 2 | 2 | 1 | 4 |
| | Tumen SAU | 0 | 2 | 1 | 0 | 7 |
| | Average | 0.50 | 1.25 | 1.50 | 0.50 | 6.25 |
| 2021-22 | Chelyabinsk ARI | 0 | 4 | 0 | 0 | 6 |
| | Kurgan Seeds | 0 | 3 | 0 | 1 | 6 |
| | Omsk SAU | 0 | 2 | 0 | 0 | 8 |
| | Tumen SAU | 0 | 4 | 0 | 0 | 6 |
| | Average | 0 | 3.25 | 0 | 0.25 | 6.50 |
| 2023-24 | Chelyabinsk ARI | 2 | 1 | 1 | - | 6 |
| | Kurgan Seeds | 1 | 0 | 1 | - | 8 |
| | Omsk SAU | 2 | 1 | 1 | - | 6 |
| | Tumen SAU | 2 | 1 | 0 | - | 7 |
| | Average | 1.75 | 0.75 | 0.75 | - | 6.75 |
